# Medical Treatment and Weight Gain

**Published:** 2007-06-15

**Authors:** Eileen McGinn

**Affiliations:** Student, Hunter College of the City University of New York, Brookdale Center for Healthy Aging and Longevity, New York, NY

## To the Editor:

"Addressing the Obesity Epidemic: A Genomics Perspective" in your April 2007 (1) issue speaks of the importance of considering all aspects of obesity. However, one aspect overlooked in the article is weight gain associated with medical treatment. Many classes of drugs are associated with weight gain that leads to overweight or obesity; these include atypical antipsychotic drugs, lithium, some antidepressant drugs, some antiepileptic drugs, and some steroids. Other drugs are associated with fat redistribution (e.g., some drugs for HIV/AIDS).

In addition to increasing weight, atypical antipsychotic drugs increase risk for hyperglycemia (as noted in a black-box label required by the Food and Drug Administration [FDA]), and they are associated with lipid dysregulation (2). Atypical antipsychotic drugs induce excitation and hypomania or mania, adverse effects never reported for the older versions of typical antipsychotic drugs (3). Some atypical antipsychotic drugs are also noncardiac QTc-interval–prolonging drugs and are associated with increased sudden cardiac death (4).

Atypical antipsychotic drugs are widely used off-label. The FDA issued a Public Health Advisory warning of a 60% to 70% increased risk for mortality among elderly people with dementia being treated with atypical antipsychotic medications (5). From 1993 through 2002, prescriptions for atypical antipsychotic drugs for American children increased 500% (all off-label); 85% of those prescriptions were for nonpsychotic conditions (6). The public health implications of wide off-label use of this class of drugs merits more study.

**Read the author's reply**
